# Sir E. L. Bulwer and Hydropathy

**Published:** 1847-08

**Authors:** 


*Sir E. L. Bulwer and Hydropathy.*—Sir E. L. Bulwer, the novelist, who not long since wrote a romance, professedly founded on facts of his personal history, in praise of Hydropathy, has, as stated by the Annalist, returned to the true faith. Will he take pains to correct the evil he may have done by his former precipitate publication? We trow not. Like most others he will rest content with availing himself of the benefits of legitimate medicine, while his philanthropic efforts to bring the Medical profession into discredit and contempt, will remain unretracted, and unatoned for. Our readers will perhaps recollect that we predicted his new faith would be of short duration.

